# Predicting complete loss to follow-up after a health-education program: number of absences and face-to-face contact with a researcher

**DOI:** 10.1186/1471-2288-11-145

**Published:** 2011-10-27

**Authors:** MJ Park, Yoshihiko Yamazaki, Yuki Yonekura, Keiko Yukawa, Hirono Ishikawa, Takahiro Kiuchi, Joseph Green

**Affiliations:** 1 Graduate School of Medicine, The University of Tokyo, 7-3-1 Hongo, Bunkyo-ku, Tokyo 113-0033, Japan

## Abstract

**Background:**

Research on health-education programs requires longitudinal data. Loss to follow-up can lead to imprecision and bias, and *complete *loss to follow-up is particularly damaging. If that loss is predictable, then efforts to prevent it can be focused on those program participants who are at the highest risk. We identified predictors of complete loss to follow-up in a longitudinal cohort study.

**Methods:**

Data were collected over 1 year in a study of adults with chronic illnesses who were in a program to learn self-management skills. Following baseline measurements, the program had one group-discussion session each week for six weeks. Follow-up questionnaires were sent 3, 6, and 12 months after the baseline measurement. A person was classified as completely lost to follow-up if none of those three follow-up questionnaires had been returned by two months after the last one was sent.

We tested two hypotheses: that complete loss to follow-up was directly associated with the number of absences from the program sessions, and that it was less common among people who had had face-to-face contact with one of the researchers. We also tested predictors of data loss identified previously and examined associations with specific diagnoses.

Using the unpaired t-test, the U test, Fisher's exact test, and logistic regression, we identified good predictors of complete loss to follow-up.

**Results:**

The prevalence of complete loss to follow-up was 12.2% (50/409). Complete loss to follow-up was directly related to the number of absences (odds ratio; 95% confidence interval: 1.78; 1.49-2.12), and it was inversely related to age (0.97; 0.95-0.99). Complete loss to follow-up was less common among people who had met one of the researchers (0.51; 0.28-0.95) and among those with connective tissue disease (0.29; 0.09-0.98). For the multivariate logistic model the area under the ROC curve was 0.77.

**Conclusions:**

Complete loss to follow-up after this health-education program can be predicted to some extent from data that are easy to collect (age, number of absences, and diagnosis). Also, face-to-face contact with a researcher deserves further study as a way of increasing participation in follow-up, and health-education programs should include it.

## Background

Studies of health-education programs require that enough data be collected at the right times [1]. However, in most longitudinal studies some loss to follow-up is considered to be inevitable and it can cause imprecision and bias [2-6].

To increase precision, one option for both observational and experimental designs is to inflate the target sample size to compensate in advance for the expected loss [7-10]. Better still, some data loss can be prevented. Here we are concerned with follow-up data collected via postal questionnaires. Among the options that have been used to promote retention in this context are recorded delivery, monetary incentives, and use of handwritten addresses [11]. If loss to follow-up can be predicted, that is, if individuals who are unlikely to return questionnaires can be identified before those questionnaires are sent, then researchers will know on whom to focus their efforts to promote retention.

Problems caused by missing data can sometimes be mitigated with statistical techniques [12]. However, those techniques are less useful when, in the worst cases, some program participants do not respond to any requests for follow-up information, so it is particularly important to predict *complete *loss to follow-up. Our goal was to identify predictors of complete loss to follow-up in a longitudinal cohort study of people who participated in a health-education program, as a step toward increasing retention and reducing bias and imprecision in future studies. We tested hypotheses regarding two potential predictors: the number of absences, and face-to-face contact with a researcher.

## Methods

Data were collected as part of a longitudinal study in Japan of adults with chronic illnesses who joined a program to learn self-management skills [13]. The program comprised group-discussion sessions with two lay leaders, and there was one session each week for six consecutive weeks. The program was open to women and men equally. Participation in the program and in this research were voluntary. This study was approved by the Research Ethics Committee of the Graduate School of Medicine at the University of Tokyo.

Before the first group-discussion session, informed consent was obtained in writing and data were collected with a questionnaire. This baseline questionnaire asked about age, schooling, marital status, and diagnoses. It also had questions asking about health status, health-related behaviors, and psychological factors, including self-efficacy for self-management of chronic disease.

The program was organized and administered by the Japan Chronic Disease Self-Management Association. That association invited researchers from the University of Tokyo (faculty and postgraduate students) to the first session for each discussion group. Thus, the people in some of the groups were introduced to one of the researchers, who explained the study and then distributed the informed-consent form and the baseline questionnaire. After distributing them, the researcher waited, and then collected them. The first baseline questionnaires were completed in August 2006. However, because the sessions were held outside Tokyo, this required at least one postgraduate student to be away for at least two days every time a new discussion group was organized, so the research team decided (in February 2008) to try sending the informed-consent forms and baseline questionnaires by postal mail instead. Those documents were then sent (together with a self-addressed post-paid envelope) and returned by postal mail about two weeks before the first group-discussion session. Researchers were not among the leaders of the discussion groups, and thus the people who received the informed-consent forms and baseline questionnaires by postal mail had no face-to-face contact with a researcher.

Follow-up questionnaires were sent by postal mail 3, 6, and 12 months later. A self-addressed post-paid envelope was included. If a follow-up questionnaire was not returned within two weeks, a reminder postcard was sent. The postcards were preprinted and then signed by hand (by MJP). A person was classified as completely lost to follow-up if none of the three follow-up questionnaires had been returned by two months after the last one was sent. The last follow-up questionnaires were sent in December 2010.

We tested the hypothesis that complete non-participation in follow-up was directly related to another form of non-participation: absence, defined as the number of group-discussion sessions not attended. Also, because some of the baseline questionnaires were distributed in person by a researcher and others were sent by postal mail, we were able to test the hypothesis that complete loss to follow-up was more common among people who had not had face-to-face contact with one of the researchers.

In addition, we quantified associations with predictors of attrition or missing data that have been studied previously, though in other countries and in different clinical contexts: self-efficacy [14,15], multimorbidity [16-18], diagnosis of depression [17], sex [16,19-21], age [16,19-23], schooling [16,23], and marital status [16,19,20], and we examined associations with other diagnoses (allergic disease, asthma, cancer, cardiovascular disease, connective tissue disease, diabetes, fibromyalgia syndrome, pulmonary disease, rheumatic disease, and vascular disease).

To analyze the data we used IBM SPSS version 19. As preliminary bivariate screening tests, for each categorical variable we used Fisher's exact test and for each continuous variable we used the unpaired t-test or the Mann-Whitney U test. Then, using the predictors with *P *< 0.05 from those tests we also did logistic regression analyses, including multivariate analysis (*P *values are listed in the supplementary table in Additional file 1).

Predictors can also be evaluated in terms of their sensitivity and specificity, and the area under the receiver operating characteristic (ROC) curve. Those indices are commonly used to evaluate predictive models [24,25], and they have been used previously to evaluate predictors of attrition in longitudinal studies of health-related interventions and educational programs [26-32]. They also provide a basis for estimating how many of the people who would otherwise be lost to follow-up could instead be identified beforehand if a predictor is used. The maximum values of sensitivity, specificity, and the area under the ROC curve are all 1, and, in general, better predictors have higher values. Introductions to this topic can be found on the Internet [33] and in reference 34 [34], and more details about the use of sensitivity, specificity, and ROC curves in the analysis of predictors can be found in references 24 [24], 25 [25], and 35 [35].

## Results

Among 409 people in 78 discussion groups who filled out the baseline questionnaire, 206 (in 31 groups) received and returned it in person and 203 (in 47 groups) received and returned it by postal mail. Results for the study participants as a whole are shown in Table 1. The prevalence of complete loss to follow-up was 12.2% (50/409).

**Table 1:** Demographic and clinical characteristics of the group as a whole (n = 409)

		**Number (%)**	
		
Sex	Male	84	(20.5)
	Female	325	(79.5)
Age	Mean (range)	47.4	(18 - 83)
Schooling	High school or less	201	(49.1)
	College or more	208	(50.9)
Marital status	Living together	215	(52.6)
	Others	194	(47.4)
Diagnoses*	Allergic disease	105	(25.7)
	Connective tissue disease	67	(16.4)
	Diabetes	65	(15.9)
	Vascular disease	65	(15.9)
	Rheumatic disease	47	(11.5)
	Fibromyalgia syndrome	32	(7.8)
	Cardiovascular disease	24	(5.9)
	Cancer	23	(5.6)
	Asthma	21	(5.1)
	Depression	17	(4.2)
	Pulmonary disease	13	(3.2)
Number of diagnoses	1	240	(58.7)
	2	98	(24.0)
	3	46	(11.2)
	≥ 4	25	(6.1)
Number of absences	0	198	(48.4)
	1	95	(23.2)
	2	52	(12.7)
	3	22	(5.4)
	4	19	(4.6)
	5	19	(4.6)
	6	4	(1.0)

* Includes multiple diagnoses.

Compared with the people who returned at least one follow-up questionnaire, those who were completely lost to follow-up had more absences and were younger (Tables 2 and 3). They were also less likely to have met one of the researchers. The percentages of people who were lost to follow-up from the "face-to-face contact: yes" and "face-to-face contact: no" groups were, respectively, 9% and 16% (18/206 and 32/203). Those having a diagnosis of connective tissue disease were less likely to be lost to follow-up than those not having connective tissue disease, and this predictor was very sensitive, though nonspecific.

**Table 2:** Analyses of predictors of complete loss to follow-up.

Predictor variable		Lost to follow-up n = 50	Not lost to follow-up n = 359	Odds ratio (95% confidence interval)	Area under ROC curve, or sensitivity and specificity
**Hypothesized predictors**					
Number of absences^a^	Median (25%, 75%)	2.0 (0, 5)	0 (0, 1)	1.78 (1.49-2.12)	0.723
	95% CI	1 to 4	0 to 1		
Contact^b^	Yes	18	188	0.51 (0.28-0.95)	0.64, 0.52
	No	32	171		
**Other analyses^c^**					
Age^d^	Mean ± SD	42.5 ± 14.1	48.1 ± 14.0	0.97 (0.95-0.99)	0.623
	95% CI	38.6 to 46.4	46.6 to 49.5		
Sex	Female	40	285	1.04 (0.50-2.17)	0.20, 0.79
	Male	10	74		
Schooling^e^	High	23	185	0.80 (0.44-1.44)	0.54, 0.52
	Low	27	173		
Marital status^f^	Together	21	194	0.61 (0.34-1.11)	0.58, 0.54
	Not together	29	164		
Self-efficacy^g^	Mean ± SD	32.2 ± 12.5	32.2 ± 12.2	1.00 (0.98-1.02)	0.502
	95% CI	28.7 to 35.7	31.0 to 33.5		
> 3 diagnoses	With	1	24	0.29 (0.04-2.15)	0.98, 0.07
	Without	49	335		
Allergic disease	With	15	90	1.28 (0.67-2.45)	0.30, 0.75
	Without	35	269		
Connective tissue disease	With	3	64	0.29 (0.09-0.98)	0.94, 0.18
	Without	47	295		
Diabetes	With	6	59	0.69 (0.28-1.70)	0.88, 0.17
	Without	44	300		
Vascular disease	With	10	55	1.38 (0.65-2.93)	0.20, 0.85
	Without	40	304		
Rheumatic disease	With	4	43	0.64 (0.22-1.86)	0.92, 0.12
	Without	46	316		
Fibromyalgia syndrome	With	5	27	1.37 (0.50-3.73)	0.10, 0.92
	Without	45	332		
Cardiovascular disease	With	0	24	0^h^	1.00, 0.07
	Without	50	335		
Cancer	With	2	21	0.67 (0.15-2.95)	0.96, 0.06
	Without	48	338		
Asthma	With	3	18	1.21 (0.34-4.26)	0.06, 0.95
	Without	47	341		
Depression	With	4	13	2.31 (0.72-7.40)	0.08, 0.96
	Without	46	346		
Pulmonary disease	With	3	10	2.23 (0.59-8.39)	0.06, 0.97
	Without	47	349		

For the number of absences, the table shows medians, 25th & 75th percentiles, and 95% confidence intervals.

For the other two continuous variables, the table shows means ± SDs, 95% confidence intervals, and areas under ROC curves.

For each categorical variable, the table shows numbers of people in each category, odds ratio and its 95% confidence interval, sensitivity, and specificity.

^a ^Number of absences from program sessions; minimum = 0, maximum = 6. Odds ratio from simple logistic regression.

^b ^No: did not have in-person contact with one of the researchers; yes: did have contact, once, at the time baseline data were collected.

^c ^Analyses of predictors studied previously or suggested during peer review.

^d ^Age in years. Odds ratio from simple logistic regression.

^e ^Low: high school or less; high: college or more.

^f ^Together: living together; not: all others

^g ^Score on a 0-to-60 scale measuring self-efficacy to manage symptoms; odds ratio from simple logistic regression.

^h ^Because no people with cardiovascular disease were completely lost to follow-up, there was complete separation in the "complete loss to follow-up" category. For a conservatively-biased estimate, changing the 0 to 1 and the 50 to 49 would yield an odds ratio of 0.29 (0.04-2.15).

**Table 3:** Simple and multiple logistic-regression models (dependent variable: complete loss to follow-up)

Independent variables^a^	Coefficient (β)	Standard error	Wald χ^2^	*P *value	Odds ratio^b^	ROC curve area
**Four models, each with one independent variable**						

Intercept	-2.91	0.25	-	-	-	
Number of absences	0.58	0.09	41.54	< 0.001	1.78 (1.49-2.12)	0.723
Intercept	-1.68	0.19	-	-	-	
Contact	-0.67	0.31	4.58	0.032	0.51 (0.28-0.95)	0.582
Intercept	-0.65	0.52	-	-	-	
Age	-0.03	0.01	6.59	0.010	0.97 (0.95-0.99)	0.623
Intercept	-3.06	0.59	-	-	-	
Connective tissue disease	-1.22	0.61	4.01	0.045	0.29 (0.09-0.98)	0.559

**One model with three independent variables**						0.752^c^
Intercept	-1.60	0.61	-	-	-	
Number of absences	0.55	0.09	35.58	< 0.001	1.73 (1.44-2.07)	
Contact	-0.59	0.34	3.02	0.083	0.56 (0.29-1.08)	
Age	-0.02	0.01	3.49	0.062	0.98 (0.96-1.00)	

**One model with four independent variables**						0.771^c^
Intercept	-1.31	0.63	-	-	-	
Number of absences	0.54	0.09	33.42	< 0.001	1.72 (1.43-2.06)	
Contact	-0.73	0.34	4.53	0.033	0.48 (0.25-0.94)	
Age	-0.02	0.01	3.81	0.051	0.98 (0.95-1.00)	
Connective tissue disease	-1.40	0.64	4.73	0.030	0.25 (0.07-0.87)	

^a ^All variables are defined as in Table 2.

^b ^Values in parentheses show 95% confidence intervals. For the models with more than one independent variable, adjusted odds ratios are shown.

^c ^This ROC area applies to the full multivariate model.

Loss to follow-up was not associated with sex, schooling, marital status, self-efficacy, having more than three diagnoses, having a diagnosis of depression, or having any of the other nine diagnoses.

When the cutoff for predicting complete loss to follow-up was set at two or more absences, the sensitivity and specificity were, respectively, 0.62 and 0.76. Thus, 62% of the 50 people who ultimately were completely lost to follow-up could have been identified no later than the time when the last group-discussion session ended, which was six weeks before the first follow-up questionnaire was sent. Predictions based on not having met one of the researchers were less specific though they were slightly more sensitive (sensitivity and specificity: 0.64 and 0.52).

**Figure 1:**
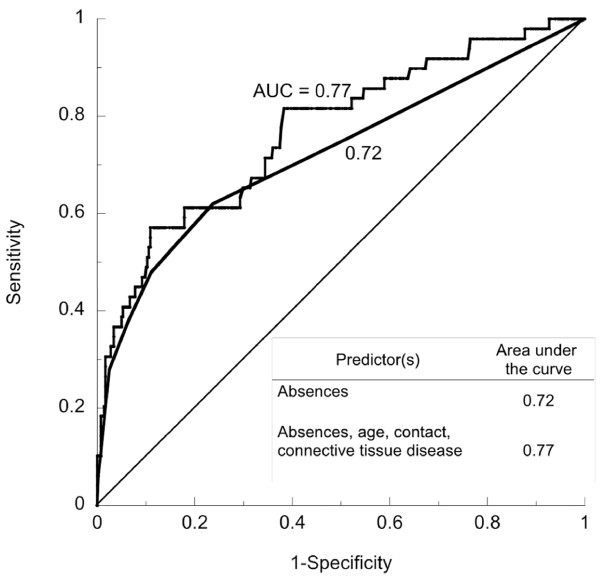
**ROC curves for two predictors of complete loss to follow-up**. One predictor is the number of absences (area under the curve = 0.72), and the other is a logistic regression model with four independent variables (number of absences, age, face-to-face contact with a researcher, and connective tissue disease; area under the curve = 0.77).

One multivariate model (Table 3) had three independent variables: the number of absences, face-to-face contact with a researcher, and age. The other model also included connective tissue disease (Table 3 and Figure 1). As predictors, they were better than the model with the number of absences alone (Table 3). For the model with four independent variables, with the cutoff set at a probability of 0.83, the sensitivity was 0.82 and the specificity was 0.62.

## Discussion

The number of absences was a good predictor of complete loss to follow-up. This supports the hypothesis that one form of non-participation could be used to predict another. Aubrey et al. reported a similar finding regarding participation in psychological therapy: early non-attendance could be used to predict attrition [36]. The other good predictors were age, face-to-face contact with a researcher, and the diagnosis of connective tissue disease. For those latter three, the associations were negative, that is, loss to follow-up was more common among people who were younger, those who had no face-to-face contact with a researcher, and those who did not have connective tissue disease.

The results regarding the number of absences lead to specific recommendations for future studies. Administrators of this health-education program and of others similar to it, and researchers studying those programs, should keep records of group-discussion attendance, and they should use those records to identify people who are at high risk of loss to follow-up. In the present case, if "two or more absences" had been used as a predictor, then 31 people who ultimately were completely lost to follow-up could have been identified beforehand (0.62 × 50 = 31). Age and diagnoses should also be recorded, so a multivariate model can be used to increase that number further. Whether the predictor is a multivariate model or the number of absences alone, all of the data needed are available six weeks before the first follow-up questionnaire is sent. Once people who are at high risk of loss to follow-up are identified, at least some of the methods known to be effective [11] should be used to try to retain them in the study.

The results regarding face-to-face contact with a researcher are also noteworthy, because that is the only predictor in Table 2 that is modifiable. Meta-analysis has shown that pre-notification by telephone is more effective than pre-notification by postal mail for increasing the initial response rates to postal questionnaires (analysis 62.1 in reference 11 [11]), but not the final response rates (analysis 62.2 in reference 11 [11], although the results are very heterogeneous: I^2 ^= 85%). We found no previous studies of the effects of face-to-face contact with a researcher on subsequent rates of postal questionnaire return. In the present study, people who had such contact immediately before they filled out the baseline questionnaire were less likely to be lost to follow-up over the following year. The implication for research is that such contact should be used as an independent variable in randomized controlled trials with participation in follow-up as the outcome. The implication for practice is that researchers should try to meet and talk with the people to whom they will later send follow-up postal questionnaires.

Such face-to-face contact will probably not be free of charge. The cost and the availability of funds to cover it will of course depend on local circumstances, though a reasonable generalization might be that face-to-face contact will be less costly in studies with fewer participants. In those situations its benefit would also be greater, because small studies can least afford the loss of precision caused by even small absolute numbers of missing data and the resulting bias if those data are not missing at random (for example, preventing 10 losses to follow-up in a study of 50 people is more beneficial than preventing 10 losses to follow-up in a study of 500 people).

Another point to consider is the fact that many more women than men were in this study. Although the present results might not apply to a program with a much smaller percentage of women, such programs seem to be rare, while programs with many more women than men are typical. In 17 studies of programs such as this one (i.e., focusing on self-management of chronic illness [10,37-52]), the percentage of women participants ranged from 61.1% [42] to 88.9% [44] and the mean was 75.7%. In the present study it was 79.5%.

Some limitations of this study should be kept in mind. We cannot be sure why people were absent or why questionnaires were not returned. Death is one possible explanation, but it is not likely, given the facts that the follow-up period was only 1 year, that those lost to follow-up were relatively young, and that they were no more likely than the others to have high multimorbidity. Change of address is also not a likely explanation. In Japan the post office forwards mail for 1 year, after which it is returned to the sender undelivered, but no questionnaires or reminder postcards were returned undelivered. Using the number of absences to predict loss to follow-up will be most beneficial if efforts are also made to find out each person's reasons for absence and for not returning follow-up questionnaires, so the type of missing data can be identified for each outcome of interest [2,12]. We also note that questions remain about the generalizability of the results across countries, programs, and types of surveys.

## Conclusions

Even with the limitations mentioned above, the potential for bias and for loss of precision make it important to predict and prevent non-participation in follow-up. The present findings lead us to specific recommendations: First, face-to-face contact with a researcher deserves further study as a way of increasing participation in follow-up, and studies of these programs should include it. Second, particularly for research on these kinds of programs, one or more of the other predictors in a multivariate model (all of which are known before the first follow-up questionnaire is sent) should be used to identify people who are not likely to return follow-up questionnaires. Once identified, those people should receive special attention [11] to promote questionnaire return.

## Competing interests

The authors declare that they have no competing interests.

## Authors' contributions

MJP contributed to conceiving and designing the study, collecting, entering, and analyzing the data, interpreting the results, and writing and revising the manuscript. YYa obtained funding for the study, and contributed to designing the study and revising the manuscript. YYo contributed to designing the study, collecting the data, and revising the manuscript. KY contributed to designing the study, collecting the data, and revising the manuscript. HI contributed to analyzing the data, interpreting the results, and revising the manuscript. TK contributed to analyzing the data, interpreting the results, and revising the manuscript. JG contributed to conceiving and designing the study, interpreting the results, and writing and revising the manuscript. All authors read and approved the final manuscript.

## Pre-publication history

The pre-publication history for this paper can be accessed here:

http://www.biomedcentral.com/1471-2288/11/145/prepub

## Supplementary Material

Additional file 1 **Supplementary table**. *P *values used in screening for variables to be included in multivariate analysis. Click here for file
