# Supplementary material for: Predicting complete loss to follow-up after a health-education program: number of absences and face-to-face contact with a researcher
Source: BMC Med Res Methodol. 2011 Oct 27;11:145. doi: 10.1186/1471-2288-11-145 (PMC3215183; doi:10.1186/1471-2288-11-145)
Supplement: Additional file 1 — Supplementary table. P values used in screening for variables to be included in multivariate analysis. [file 1471-2288-11-145-S1.DOC]

| Supplementary table: *P* values used in screening for variables to be included in multivariate analysis. | |
| --- | --- |
| Predictor | *P* value^a^ |
|  |  |
| **Hypothesized predictors** |  |
| Number of absences | < 0.001 |
| Contact | 0.035 |
|  |  |
| **Other analyses**^b^ |  |
| Age | 0.009 |
| Sex | > .999 |
| Schooling | 0.546 |
| Marital status | 0.130 |
| Self-efficacy | 0.982 |
| > 3 diagnoses | 0.340 |
| Allergic disease | 0.490 |
| Connective tissue disease | 0.040 |
| Diabetes | 0.537 |
| Vascular disease | 0.410 |
| Rheumatic disease | 0.635 |
| Fibromyalgia syndrome | 0.572 |
| Cardiovascular disease | 0.058 |
| Cancer | > .999 |
| Asthma | 0.732 |
| Depression | 0.141 |
| Pulmonary disease | 0.204 |
|  |  |
|  |  |
| ^a^ Mann-Whitney U test for number of absences, unpaired t-test for age and for self-efficacy, and Fisher's exact test for all others. | |
| ^b^ Predictors studied previously or suggested during peer review. | |
